# Interface second harmonic generation enhancement in bulk WS_2_/MoS_2_ hetero-bilayer van der Waals nanoantennas

**DOI:** 10.1038/s41377-025-01983-y

**Published:** 2025-09-29

**Authors:** Andrea Tognazzi, Paolo Franceschini, Jonas Biechteler, Enrico Baù, Alfonso Carmelo Cino, Andreas Tittl, Costantino De Angelis, Luca Sortino

**Affiliations:** 1 Department of Engineering, University of Palermo, Viale delle Scienze, 90128 Palermo, Italy; 2 National Institute of Optics - National Research Council (INO-CNR), Via Branze 45, 25123 Brescia, Italy; 3 Department of Information Engineering, University of Brescia, Via Branze 38, 25123 Brescia, Italy; 4 Chair in Hybrid Nanosystems, Nanoinstitute Munich, Faculty of Physics, Ludwig-Maximilians-Universität München, 80539 Munich, Germany

**Keywords:** Optical materials and structures, Nonlinear optics, Nanophotonics and plasmonics

## Abstract

Layered van der Waals (vdW) materials have emerged as a promising platform for nanophotonics due to large refractive indexes and giant optical anisotropy. Unlike conventional dielectrics and semiconductors, the absence of covalent bonds between layers allows for novel degrees of freedom in designing optically resonant nanophotonic structures down to the atomic scale: from the precise stacking of vertical heterostructures to controlling the twist angle between crystallographic axes. Specifically, although monolayers of transition metal dichalcogenides exhibit giant second-order nonlinear responses, their bulk counterparts with 2H stacking possess zero second-order nonlinearity. In this work, we investigate second harmonic generation (SHG) arising from the interface of WS_2_/MoS_2_ hetero-bilayer thin films with an additional SHG enhancement in nanostructured optical antennas, mediated by both the excitonic resonances and the anapole-driven field enhancement. When both conditions are met, we observe up to 10^2^ SHG signal enhancement, compared to unstructured bilayers, with SHG conversion efficiency reaching ≈ 10^−7^. Our results highlights vdW materials as a platform for designing unique multilayer optical nanostructures and metamaterial, paving the way for advanced applications in nanophotonics and nonlinear optics.

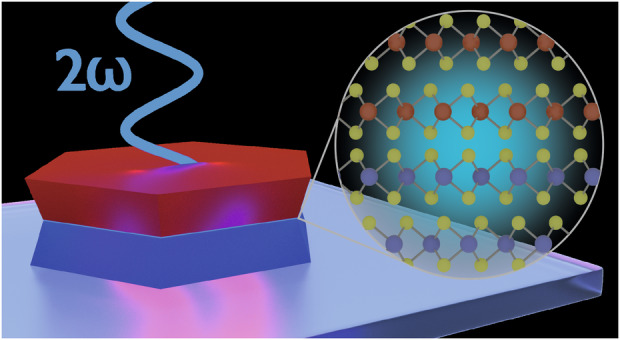

## Introduction

High refractive index dielectric materials, such as silicon, gallium phosphide, and III-V semiconductors, have emerged due to their exceptional ability to confine light within nanostructured optical resonators^1–3^. Unlike plasmonic metallic counterparts, which primarily exploit surface plasmon resonances, dielectric materials leverage Mie resonances, with both electric and magnetic components^4^. This unique characteristic allows for the exploration of new degrees of freedom in nanophotonic design, where nanoresonators can be precisely engineered to manipulate the interference between electric and magnetic resonances^5^. By tailoring the combination of material properties, geometry, and optical excitation, modulation of the directional emission and non-trivial optical states can be achieved. For instance, non-radiative dark states within resonant dielectric nanostructures^6^ result from the interference between different radiative channels, leading to strong confinement of electromagnetic energy connected with a suppression of far-field scattered radiation. In this regard, significant attention has been directed towards anapole states^7^ and bound states in the continuum resonances^8^, finding applications for linear and non-linear optics owing to the increased internal electromagnetic energy in the system and versatile control over the radiated pattern^9,10^. The fabrication of conventional dielectric nanoresonators typically relies on the growth of polycrystalline thin films, which suffer from lattice mismatch at hetero-interfaces, limiting the use of arbitrary substrates and the creation of multilayered optical structures^11^. Recently, van der Waals (vdW) materials emerged as a new class of crystals for non-linear optics and nanophotonics^12^ promising to overcome current dielectric materials’ limitations^13,14^.

Due to their crystal structure, which features strong in-plane covalent bonds and weak vdW forces between planes, vdW crystals can be mechanically exfoliated into thin crystalline layers on arbitrary substrates, down to atomic thicknesses. Moreover, the absence of covalent bonds between layers enables the deterministic stacking of multiple layers, forming so-called vdW heterostructures^15^. Owing to their remarkable optical and structural properties, atomically thin two-dimensional (2D) semiconductors, such as transition metal dichalcogenides (TMDCs), have been at the forefront of nanophotonics research in recent years, ranging from integrated components, light-matter coupling, and non-linear optics^16,17^. In this regard, second harmonic generation (SHG) has been a long standing technique for characterizing single and few TMDC layers^18^, as well as for imaging of mechanical deformations^19^, further exhibiting unconventional effects, from quantum interference^20^, to broadband phase matching^21^ and all-optical modulation^22^. Beyond their 2D form, vdW materials thin films (<100 nm in thickness) have attracted large attention as new building blocks of integrated nanophotonic structures^23–25^. They provide exciting properties for nanoscale dielectric resonators, such as large anisotropy^26^, high refractive indexes^25,27^, and wide substrate affinity^28^, along with an ever growing library of materials. Nanophotonic structures with TMDC thin films have been demonstrated, from anapole nanoantennas^28–30^ and optical metasurfaces^31–34^, to linear and non-linear waveguides^35,36^ and optical modulators^37^.

As research on TMDCs films for nanophotonics is still in its infancy, so far the exploration of TMDC optical nanostructures relied on single exfoliated materials^38^. However, vdW crystals possess the unique property of engineering second order non-linear processes at the interface between layers by tuning the symmetry and twist angle between adjacent crystal planes^39^. While variants of TMDCs exhibit strong bulk second-order susceptibility^12^, the nonlinear tensor is relatively fixed by the material symmetry and crystal structure. In contrast, vdW interfaces enable interface-specific nonlinear optical responses that are not accessible in bulk materials. This includes breaking the nonlinear tensor symmetry at the interface and emergent excitonic coupling that enhances and modulates the SHG signal beyond bulk-related effects. A study on optical resonators made with TMDCs thin film heterostructures is still missing, which could open to the control of interface driven non-linear effects^40^ and novel degrees of freedom in the design of non-linear^41^ and chiral^42^ vdW-based optical metamaterials, directional non-linear light emission^43^ and surface polaritons^44^.

In this work, we demonstrate SHG arising from the interface between two dielectric vdW materials, and its additional enhancement due to the interplay of excitonic resonances and the anapole states in dielectric nanoresonators. Double-layer optical nanoantennas were prepared from TMDCs WS_2_/MoS_2_ hetero-bilayer thin films, obtained through mechanical exfoliation and subsequent deterministic stacking. The resulting heterostructure is then processed with standard nanofabrication methods and shaped into hexagonal resonators, revealing the underlying crystal symmetry of the materials. Due to the close values of the refractive index between the two layer, the resonators act as an homogeneous dielectric medium, allowing the design of single structures sustaining non-radiating anapole states. We confirm the presence of anapole states in the fabricated sample by linear optical reflectance measurements in the near infrared region. Although bulk TMDCs do not possess broken inversion symmetry, we observe SHG only in the presence of the WS_2_/MoS_2_ interface. Moreover, a strongly enhanced SH signal is observed in anapole nanoantennas, which is driven either by the presence of exciton resonances at the second harmonic frequency (2*ω*), due to increased *χ*^(2)^ tensor elements of the material, or by the anapole state, which increases the energy density at the fundamental (*ω*) frequency. When the SH of the anapole frequency matches the excitonic resonance, we observe up to two orders of magnitude enhancement of SHG compared to a reference unstructured hetero-bilayer. While a SHG enhancement factor of 10^2^ is common in resonant nanophotonic systems, this interface-specific mechanism, distinct from conventional dielectric materials, enables SHG from multilayers 2H-TMDCs, that are otherwise centrosymmetric with zero second order nonlinear response. Furthermore, we extract the SHG conversion efficiency (*η*_*SH*_) and the nonlinear peak coefficient (*β*_*SH*_) with maximal values of 3.3 × 10^−7^ and of 4.56 × 10^−8^ *W*^−1^, respectively, comparable with established non-centrosymmetric dielectric materials.

Our results highlight the unique potential of vdW materials for designing unprecedented vertically stacked nanophotonic structures with arbitrary materials, opening to precise control over crystal thickness and orientation. TMDCs could play a critical role in advancing nonlinear optics and nanophotonics with the interplay of intrinsic excitonic states and photonic resonances, opening new avenues for optically active linear and non-linear materials with tailored optical properties, towards on-chip SHG in integrated photonics, frequency conversion for optical communication systems, and for compact quantum optics platforms. Our findings provide a pathway for understanding and utilizing interfacial nonlinear effects in vdW materials, complementing existing studies on SHG in TMDCs, with direct implications for the design and development of emerging vdW-based nanophotonic platforms.

## Results

**Fig. 1:**
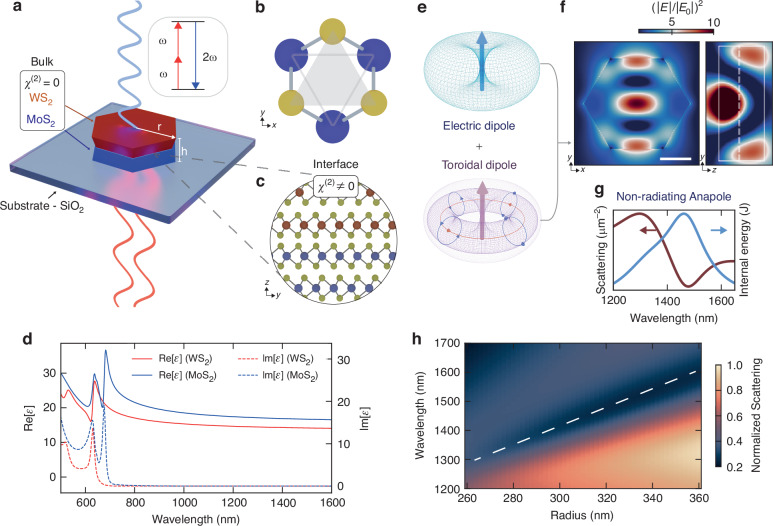
Hetero-bilayer WS_2_/MoS_2_ van der Waals hexagonal nanoantennas sustaining anapole states. **a** Schematic of the dual-layer van der Waals (vdW) nanoantenna positioned on a SiO_2_ substrate, composed of a bottom MoS_2_ layer (blue) and a top WS_2_ layer (red), each ~100-nm thick. Inset shows the second harmonic generation process where the absorption of two photons at the fundamental frequency *ω* leads to the generation of a frequency-doubled photon at 2*ω*. **b** Illustration of the in-plane TMDC crystalline honeycomb symmetry. Metal atoms are depicted in blue and chalcogenide ones in yellow. **c** Illustration of the interface between the two TMDC layers aligned at zero degrees, showing broken inversion symmetry region resulting in the second order non-linear signal. **d** Real and imaginary parts of the in-plane dielectric function for WS_2_ (red) and MoS_2_ (blue). Adapted from ref. ^27^. **e** Far field illustration of an electric dipole (top) and a toroidal dipole (bottom) whose interference generates the anapole state. **f** Numerical FDTD simulations of the electric field intensity, ${(| E| /| {E}_{0}| )}^{2}$, at the anapole wavelength for a WS_2_/MoS_2_ disk with layer thicknesses of 92 nm and 115 nm, respectively, and radius of 280 nm. The data is displayed along the *z*-plane of the WS_2_/MoS_2_ interface (left panel) and the center of the structure, along the vertical plane at *x* = 0 (right panel). Scale bar: 200 nm. **g** Numerical FDTD simulations of the normalized scattering cross section (in red) exhibiting a minima, and the normalized internal electromagnetic energy (in blue) exhibiting a maximum at the anapole wavelength. **h** Numerical FDTD simulations of the normalized scattering cross section for a WS_2_/MoS_2_ disk on a glass substrate, with radial size from 260 nm to 360 nm, and height of 92 nm for the MoS_2_ layer and 115 nm for the WS_2_ one. The sample is illuminated with normal incidence light from the air side. The dashed white line indicates the dip in far-field scattering attributed to the anapole state

Interfaces play a crucial role in breaking the inversion symmetry, a requirement for a non-zero *χ*^(2)^ tensor, overcoming materials’ restrictions and promoting the generation of second order non-linear processes even in centrosymmetric crystals. Figure 1a shows an illustration of the hetero-bilayer TMDC nanoantenna, where the interface between the two vdW materials promotes the symmetry-breaking condition for the SHG process. The dielectric nanoresonator is made of two bulk TMDCs materials, a bottom layer of MoS_2_ and a top layer of WS_2_, with an hexagonal shape owing to the selective anisotropic etching^28^. The crystal structure of a single layer of TMDCs is shown in Fig. 1b, where the honeycomb lattice leads to a non-centrosymmetric structure in single layers with *D*_3*h*_ point group^18^. However, bulk TMDCs with 2H stacking lack a broken inversion symmetry as each successive layer is rotated 180 degrees compared to the neighboring ones, resulting in a *D*_6*h*_ point group. As such, the emission of a second order non-linear signal in our sample can be ascribed to the breaking of the inversion symmetry generated at the TMDCs bulk interface (Fig. 1c). In order to maximize the efficiency of the interface SHG signal, we aligned the crystal axes to near zero-degree^25^. In Fig. 1d are shown the real and imaginary part of bulk MoS_2_ and WS_2_ used in this study. Both materials exhibit high refractive indexes in the visible and near-infrared regions. The resonance peaks observed in the visible range correspond to the excitonic states inherent to each material.

In resonant dielectric nanostructures, the interference of an electric dipole and a toroidal dipole, depicted in Fig. 1e, leads to the creation of the non-radiative anapole state, confining light and boosting non-linear optical processes^45^. Figure 1f shows the finite-difference time-domain (FDTD) numerical simulation of the electric field intensity ${(| E| /| {E}_{0}| )}^{2}$ of a WS_2_/MoS_2_ nanohexagon, where *E* is electric field amplitude of the scattered field by the antenna and *E*_0_ the normally incident field. The field exhibits the characteristic anapole field profile^7^, reaching values of one order of magnitude in field enhancement. As shown in Fig. 1g, the anapole condition results in the suppression of the far field radiation, which can be observed as a dip in the scattering cross sections. Most importantly, the resonant condition is accompanied by an increased electric field enhancement, and thus electromagnetic internal energy available for interaction with light. Figure 1h shows the FDTD simulations of the scattering cross sections for the dual layer WS_2_/MoS_2_ hexagonal antenna that we designed. The anapole scattering dip is observed to shift from ~1300 nm to 1600 nm as the radial size of the hexagonal antenna increases from 260 nm to 360 nm.

**Fig. 2:**
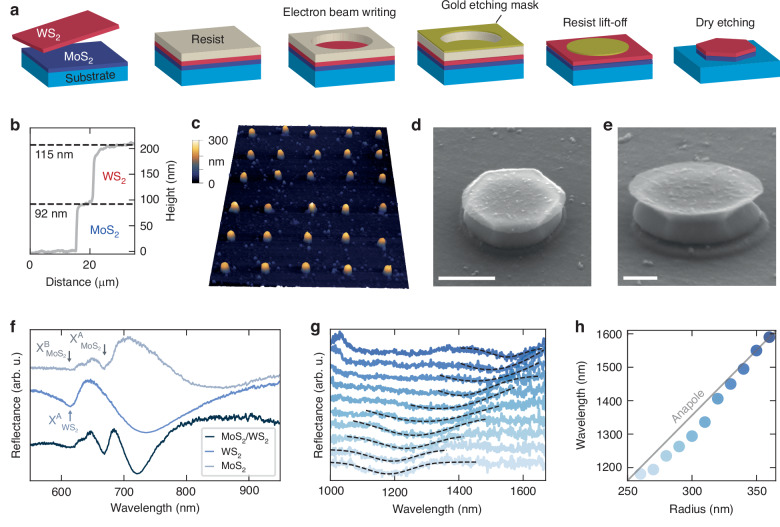
Fabrication and linear optical characterization of WS_2_/MoS_2_ anapole nanoantennas. **a** Fabrication steps of the dual TMDC layer nanoantennas. From left to right: exfoliation and transfer of the thin TMDC films, deposition of the electron beam resist, electron beam lithography writing, gold etching mask deposition, resist lift-off, and final dry etching step and mask removal (not shown). **b** Height profile of the exfoliated and stacked TMDC layers, before nanofabrication, revealing a total height of 207 nm. **c** Large area atomic force microscopy (AFM) scan of the final sample after nanofabrication. Electron microscope images of the fabricated sample, for different tilting angles of 50 (**d**) and 60 (**e**) degrees. Scale bars: 200 nm. **f** Linear reflectance spectra of the exfoliated and unpatterned reference of MoS_2_ and WS_2_, with the relative A (*X*^*A*^) and B (*X*^*B*^) exciton energies, and that of the reference double layer unpatterned stack. **g** Reflectance of a set of WS_2_/MoS_2_ antennas with radius ranging from 260 nm to 360 nm. The black dashed lines are a Gaussian fit to the relative anapole spectral dip. **h** Spectral position of the anapole condition, extracted from the fit in panel (**g**), as a function of the radius. The gray line correspond to the linear dependence predicted from numerical simulations

The fabrication steps of the double-layer nanoantennas are depicted in Fig. 2a. Starting from individual TMDCs layers being mechanically exfoliated on glass substrates from commercially available bulk single crystals. We identified large area and uniform thickness multilayers with an height of ~100 nm. The top WS_2_ layer is then transferred with a hot pick-up technique^46^ on top of the bottom MoS_2_ layer (Supplementary Fig. S1a). As exfoliation procedures do not allow a fine tuning of the layer thickness, the final heterostructure exhibit a small asymmetry in the relative height of the TMDC layers. Figure 2b shows the measured height profile of the hetero-bilayer before the nanofabrication, revealing a thickness of 92 nm (115 nm) for the MoS_2_ (WS_2_) layer. After proceeding with an electron beam lithography step and deposition of a gold etching mask, the sample is exposed to a dry etching procedure where only the exposed material is left on the substrate (Supplementary Fig. S1b). After fabrication, we characterized the sample via atomic force microscopy (AFM) and scanning electron microscopy (SEM). Figure 2c shows a large area AFM scan of an array of optical resonators after the fabrication. As further shown in Supplementary Fig. S2, the height of the fabricated nanopillars is consistent with pre-fabrication data and no changes in the TMDCs thickness is observed. We then confirm the fabrication of hexagonal nanoantennas from SEM imaging, as shown in Fig. 2d, e. For all the nanoantennas, we observe the presence of a thin (<5 nm) WS_2_ film on top of the nanoresonators, a leftover of the etching process, which is not expected to modify the optical response of the nanoresonators^29^. Moreover, the etching process yields tilted sidewalls, expected to blueshift the resonance, compared to simulations, due to a reduction of the resonator volume (see Supplementary Note III). From the etched sidewalls, the SEM images suggest the geometric alignment of the two TMDC layers close to zero angle, however, SEM does not have the resolution to confirm atomic-scale alignment or stacking order.

We characterized the sample via visible linear reflectance spectroscopy on unpatterned reference patches of the hetero-bilayer and single TMDC layers, as shown in Fig. 2f. In the reflectance spectra of the MoS_2_ and WS_2_ layers, we identified the dominant A and B exciton resonances, also present in the unpatterned WS_2_/MoS_2_ heterostructure (see also Fig. 1d). Specifically, we observed the MoS_2_ A exciton (${X}_{Mo{S}_{2}}^{A}$) at ~700 nm, and the MoS_2_ B exciton (${X}_{Mo{S}_{2}}^{B}$) along with the WS_2_ A exciton (${X}_{W{S}_{2}}^{A}$) closely resonant in energy at 630 nm. Supplementary Note IV includes the visible reflectance spectra of the fabricated nanoantennas, where these exciton signatures are also evident. Moving to the near-infrared region, we detected a dip in the reflectance of the double-layer nanostructures, indicative of the anapole state. Figure 2g presents the experimental reflectance data for a series of nanoantennas with varying radial sizes, along with the corresponding fit of the anapole dip. The dip’s spectral position reveals the expected linear dependence of the anapole wavelength on the nanoantenna radius, ranging from 1200 nm to 1600 nm (Fig. 2h), consistent with predictions from numerical simulations (see also Supplementary Figs. S3 and S4).

**Fig. 3:**
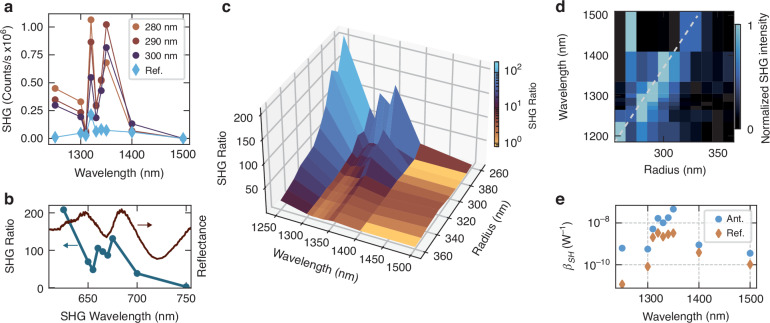
Exciton and anapole driven enhancement of WS_2_/MoS_2_ interface second harmonic generation. **a** Raw counts of the SHG intensity as a function of the fundamental wavelength, for nanoantennas with radii of 280 nm, 290 nm and 300 nm (dot markers), and the reference sample (diamond markers). **b** Comparison between the SHG ratio for a nanoantenna with radius of 280 nm and the reflectance spectrum of the reference hetero-bilayer, where the SHG enhancement is resonant with the exciton position of the TMDC layers. **c** Three-dimensional plot of the SHG ratio, as a function of the nanoantenna radius and fundamental wavelength. **d** Normalized SHG signal for the same set of nanoantennas in panel (**c**), revealing a linear dependence of the maximum SHG emission and the anapole wavelength (dashed line). **e** Nonlinear parameter (*β*_*SH*_) as a function of the fundamental excitation wavelength, shown for hetero-bilayer nanoantennas (round markers) and reference heterostructure (diamond markers)

To investigate the WS_2_/MoS_2_ nanoantennas in the non-linear regime, we excite nanoresonators of different radial size with fundamental wavelengths in the range from 1250 nm to 1500 nm employing a tunable optical parametric amplifier (see Methods for more details). The pump pulses are filtered with bandpass filters of 12 nm spectral width, and we employed a 10 nm step around the excitonic resonance energy to achieve higher resolution in this spectral range. We observe a strong SHG enhancement in the nanostructured samples (Fig. 3a) comparing the amplitude of the SH signal intensity of nanoantennas with different radii (circular markers) to the reference hetero-bilayer sample (diamond markers). The strong enhancement of the SHG is closely correlated with the TMDCs exciton resonance position (Fig. 3b). Here, we define the SHG enhancement ratio (see also Supplementary Note V) as the SHG intensity counts collected from the large area reference sample (*I*_*ref*_), normalized over the laser spot area (*A*_*laser*_), and its ratio with the SHG collected from the nanoantennas (*I*_*r*_), normalized over the relative cross sectional area (*A*_*r*_) extracted from SEM images. When the fundamental laser is resonant with ${X}_{Mo{S}_{2}}^{A}$ we observe two orders of magnitude enhancement and a peculiar double peak, replicated in both antennas and reference sample, which we ascribe to the excitonic structure of MoS_2_. We also observe even larger values at 625 nm, most likely due to the resonant ${X}_{Mo{S}_{2}}^{B}$ and ${X}_{W{S}_{2}}^{A}$ states overlapping in energy. Figure 3c shows the SHG enhancement ratio for the whole range of WS_2_/MoS_2_ nanoantennas, as a function of the radial size and fundamental excitation wavelength. In smaller radii nanoantennas, where the anapole state is also expected to be resonant with the exciton energy, we observe up to two orders of magnitude enhancement. To understand the role of the anapole field confinement on enhancing the SHG signal, we plot in Fig. 3d the normalized SHG intensity from the same set of nanoantennas. We observe that the increased SHG signal follows a linear dependence with the nanoantenna radius, matching the anapole wavelength extracted in Fig. 2g. The deviation of the maxima for 1500 nm pump wavelength is ascribed to imperfections or resonance overlaps resulting in a higher SHG intensity compared to larger radii antennas. These observations demonstrate the non-trivial interplay on the SHG emission by the combined action of excitonic bulk resonances, controlled by the choice of the layered vdW material, and the anapole field confinement, tailored via the nanostructure geometry. We can exclude edge effects on the SHG emission following the clear dependence of the SH emitted single with the field confinement provided by the anapole mode, which is confined inside the dielectric resonator. This dual influence, material selection and geometric tailoring, highlights the complexity of the system, offering deeper insights into the mechanisms governing nonlinear optical processes in multilayered vdW nanophotonic structures. Finally, we estimated the SHG conversion efficiency (*η*_*SH*_) and the nonlinear peak coefficient (*β*_*SH*_) (additional details are provided in Supplementary Note VI). In Fig. 3e, we compare the maximum *β*_*SH*_ values for the investigated nanoantennas (round markers) and for the reference pad (diamond markers) as a function of the fundamental excitation wavelength. For the interface SHG signal, we observe a maximum *β*_*SH*_ of 4.56 × 10^−8^ *W*^−1^(See Table IV in the Supplementary Note VI) and a relative value of *η*_*SH*_ = 3.3 × 10^−7^ at 1 GW cm^−2^ intensity, comparable to values reported for well-established non-centrosymmetric materials, such as AlGaAs nanopillars with *β*_*SH*_ ≈ 10^−7^ *W*^−1^ ^47^.

## Discussion

In summary, our work represents the first demonstration, to our knowledge, of using vdW heterostructures, specifically TMDCs, to fabricate optical nanoantennas to target nonlinear optical processes at the materials’ interface. We observed a significant and non-trivial SHG enhancement from the WS_2_/MoS_2_ interface, driven by the interplay of excitonic and photonic resonances of the double-layer nanoantenna. The distinct structural and optical properties of vdW materials position them as ideal candidates for future applications in nanophotonics and non-linear optics, offering new degrees of freedom in design. Moreover, unlike conventional bulk SHG crystals, the inherent anisotropies and the tunability of the twist angle between vdW crystal axes present exciting opportunities for generating nonlinear light at material interfaces which could present moiré-induced effects, with both theoretical and practical implications. Future extensions of our approach could explore spontaneous parametric down-conversion^48^ by engineering the nonlinear interface for phase-matching, optimizing directional emission via nanostructure design (e.g., via the Kerker effect), and leveraging excitonic resonances to enhance efficiency at the pump frequency. Additionally, the interplay between excitonic resonances and anapole states not only enhances SHG at the TMDC interface but also enables tunability through external factors such as strain, electric fields, or temperature, paving the way for advanced nonlinear photonic devices and entangled photon sources. For example, our approach could be integrated into waveguiding systems by embedding the SHG source, arising from the interface of the heterostructure, within vdW materials to create compact and efficient nonlinear waveguides^14^. Finally, our multilayer approach can be further extended to the broad library of vdW materials. As vdW materials enable the arbitrary stacking of different crystal structures, a deep understanding of their interfacial nonlinear properties opens to the development of engineered multilayered nanostructures and optical metamaterials optimized for enhanced light-matter interactions and non-linear optics.

## Methods

### Fabrication

WS_2_ and MoS_2_ thin films were mechanically exfoliated onto commercial silicon/silicon dioxide wafers. Flakes of suitable size and thickness were selected using optical microscopy and a profilometer (Bruker Dektak). For the fabrication of the TMDC hetero-bilayer, first the WS_2_ flake was picked up using a polydimethylsiloxane stamp with a thin layer of poly(bisphenol A carbonate) on top. The WS_2_ flake was subsequently brought in contact with the MoS_2_ flake, picked up and both components were finally stamped on a fused silica substrate. The TMDC stack was then patterned into single disk structures following the method described in reference^33^.

### Linear spectroscopy

The linear spectroscopy setup schematics is shown in Supplementary Fig. S8. We employ a tungsten lamp (Thorlabs, SLS201L) collimated by a parabolic mirror (Thorlabs, RC08FC-P01) as a white light source for both visible and NIR measurements. A polarizer (Thorlabs, GL10) and an half-waveplate (Thorlabs, AHWP10M-1600 or AHWP10M-850) controls the impinging polarization for NIR measurements. The light passes through a beam splitter (Thorlabs, BSS10R and BSN12R for visible and NIR, respectively). We use a three-axis piezo-motor stage (SmarAct) to precisely move the sample in the focal spot of the objective (Olympus, NIR 100x NA = 0.85). The reflected light is collected by a parabolic mirror (Thorlabs, RC12FC-P01) which is coupled to an optical multimode fiber connected to a visible (Andor) or NIR (Ocean Optics, NIRQuest512) spectrometer. A flip mirror routes the reflected light to a CCD camera to visualize the sample position. The normalization procedure and background subtraction is performed according to ref. ^49^, where we use the silica substrate as a reference instead of a silver mirror.

### Nonlinear spectroscopy

The nonlinear spectroscopy setup schematics is shown in Supplementary Fig. S9. We feed a Monaco laser (Coherent) at 1035 nm with 300 fs pulses and a 500 kHz repetition rate to a commercial optical parametric amplifier (Coherent, Opera-F) to tune the output wavelength between 1200 nm and 2000 nm. The output radiation is filtered by a longpass pass filter (Thorlabs, FELH1100) to remove the residual pump and a bandpass filter to select the desired wavelength (Thorlabs, FB1XX0-12 series) with a 12 nm bandwidth. The fundamental beam power is controlled by an half-wave plate (Thorlabs, AHWP10M-1600) and a polarizer (Thorlabs, GL10). The input polarization can be changed by means of an additional half-wave plate. The fundamental laser beam is transmitted by a dichroic mirror (Thorlabs, DMLP950) and then focused by a large numerical aperture objective (Olympus, NIR 100x NA = 0.85). The collected second harmonic is reflected by the dichroic mirror and filtered by a shortpass filter (Thorlabs, FESH800) before being focused (Thorlabs, LA1433) on a single photon avalanche detector (MPD, PD-50). A flip mirror and a system with a LED and a visible camera allow to image the samples. The actual laser spot size at the fundamental wavelength is determined by performing knife edge measurements.

## Supplementary information

Supplementary Information

## Data Availability

The data that support the findings of this study are available from the corresponding authors upon reasonable request.
