# Supplementary material for: Interface second harmonic generation enhancement in bulk WS2/MoS2 hetero-bilayer van der Waals nanoantennas
Source: Light Sci Appl. 2025 Sep 29;14:346. doi: 10.1038/s41377-025-01983-y (PMC12477290; doi:10.1038/s41377-025-01983-y)
Supplement: Supplementary file 1 — Supplementary Information [file 41377_2025_1983_MOESM1_ESM.pdf]

Supplementary information for:
Interface second harmonic generation enhancement in bulk WS_2/MoS_2 hetero-bilayer van der Waals nanoantennas

Andrea Tognazzi§,[1,2,*] Paolo Franceschini§,[2,3,†] Jonas Biechteler,[4] Enrico Baù,[4] Alfonso Carmelo Cino,[1] Andreas Tittl,[4] Costantino De Angelis,[2,3] and Luca Sortino[4,‡]

[1] *Department of Engineering, University of Palermo, Viale delle Scienze, 90128, Palermo, Italy*

[2] *National Institute of Optics - National Research Council (INO-CNR), Via Branze 45, 25123, Brescia, Italy*

[3] *Department of Information Engineering, University of Brescia, Via Branze 38, 25123, Brescia, Italy*

[4] *Chair in Hybrid Nanosystems, Nanoinstitute Munich, Faculty of Physics, Ludwig-Maximilians-Universität München, 80539 Munich, Germany*

CONTENTS

Supplementary Note I: Optical images of the double-layer TMDC nanoantennas 2

Supplementary Note II: AFM height profiles 2

Supplementary Note III: Linear simulations of hexagonal nanostructures 3

Supplementary Note IV: Linear visible reflectance of fabricated nanoantennas 5

Supplementary Note V: SHG normalization 6

Supplementary Note VI: Evaluation of the SHG conversion efficiency and nonlinear coefficient 7

Supplementary Note VII: linear microscopy setup 10

Supplementary Note VIII: nonlinear second harmonic generation microscopy setup 10

References 10

SUPPLEMENTARY NOTE I: OPTICAL IMAGES OF THE DOUBLE-LAYER TMDC NANOANTENNAS

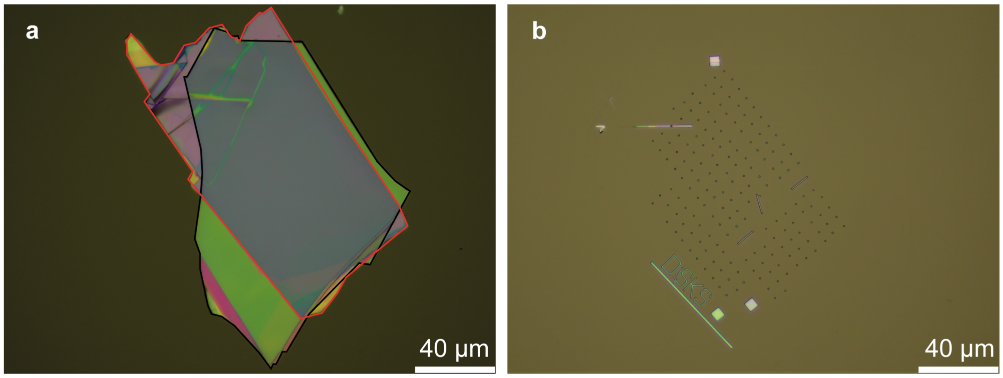

FIGURE S 1. (a) Bright field microscopy image of the fabricated stack before nanofabrication. (b) Image of the final sample after nanofabrication.

SUPPLEMENTARY NOTE II: AFM HEIGHT PROFILES

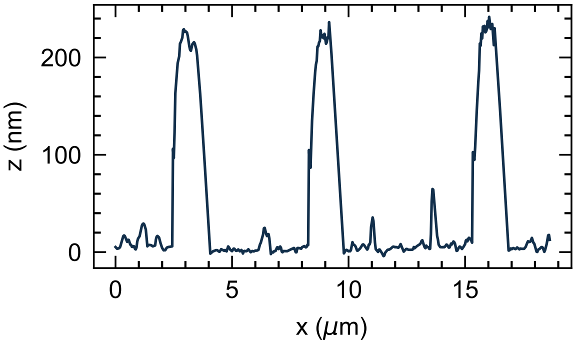

FIGURE S 2. Atomic force microscopy profiles of three fabricated nanoantennas. The thickness of the final nanostructures is consistent with the pre-fabrication observations, confirming that the overetching has not affected the height of the TMDC hetero-bilayer.

SUPPLEMENTARY NOTE III: LINEAR SIMULATIONS OF HEXAGONAL NANOSTRUCTURES

We verify that the spectral position of the anapole is not dependent upon the impinging polarization angle θ by performing linear simulations with Comsol Multiphysics as shown in Figure S3, with the dielectric constant shown in Figure 2 in the main text. We illuminate the nanoantenna with a normally incident monochromatic wave and ensure to remove any reflection from the domain boundaries by introducing perfectly matched layers and scattering boundary conditions. Although the electric field distribution is perturbed when the polarization angle is changed (see Figure S4) the spectral position of the anapole condition is not significantly affected.

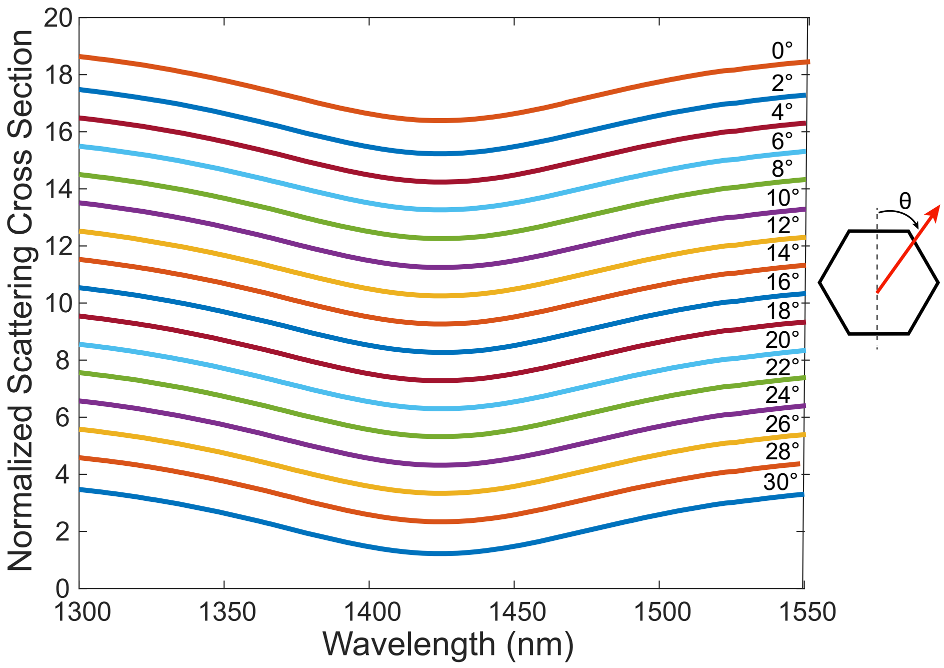

FIGURE S 3. Simulated normalized scattering cross section at different polarization angles (θ) of a disk with radius of 300 nm. The red arrow in the right sketch represents the polarization direction. The traces are vertically shifted for sake of visualization.

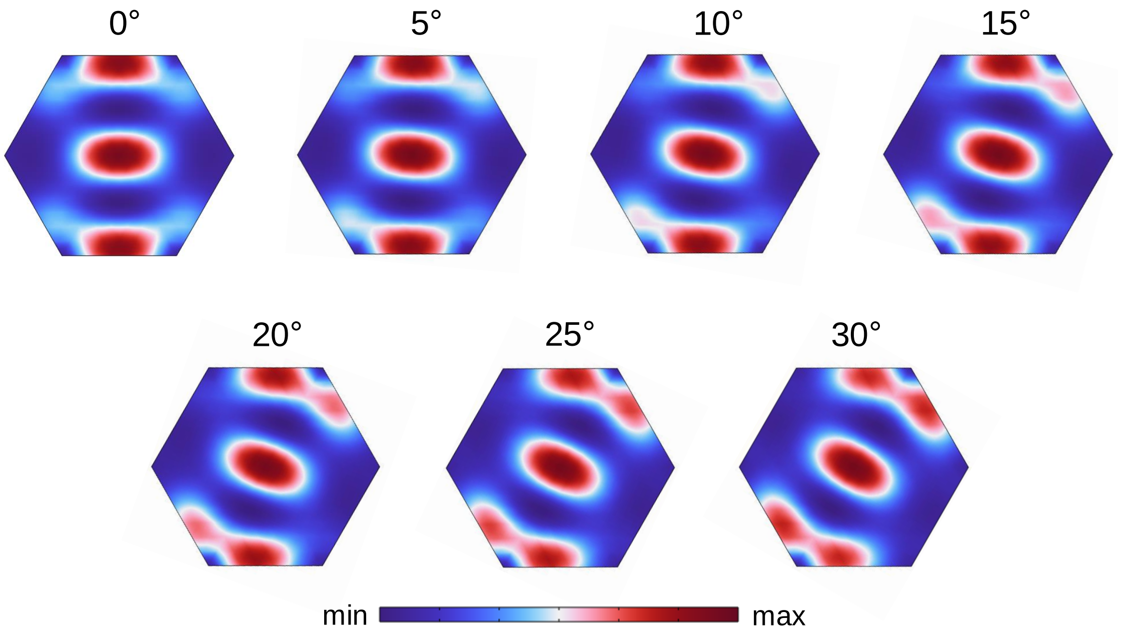

FIGURE S 4. Simulated electromagnetic field enhancement $(|E|/|E_0|)^2$ at different polarization angles at 1420 nm for a disk with radius 300 nm.

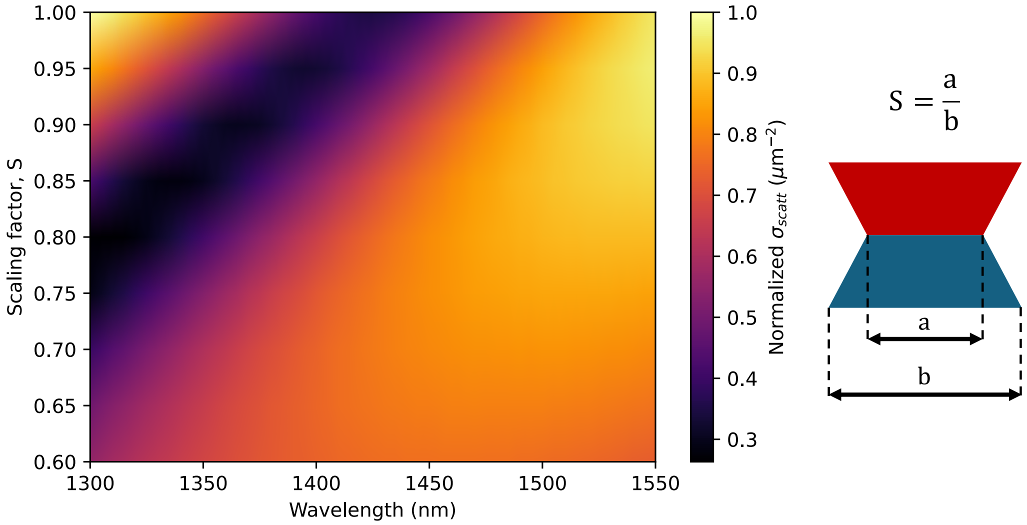

FIGURE S 5. Simulated normalized scattering cross section of tilted sidewalls nanoantennas, as a function of the wavelength and the scaling factor ($b = 300$ nm).

SUPPLEMENTARY NOTE IV: LINEAR VISIBLE REFLECTANCE OF FABRICATED NANOANTENNAS

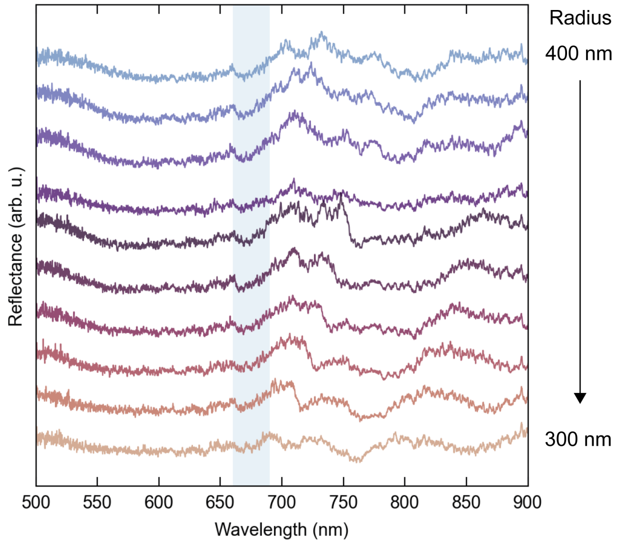

FIGURE S 6. Visible reflectance of WS_2/MoS_2 nanoantennas with radius ranging from 400 nm to 300 nm. The shaded blue area indicates the position of the $X^{\mathrm{A}}_{\mathrm{MoS_2}}$ exciton resonance.

SUPPLEMENTARY NOTE V: SHG NORMALIZATION

The experimental approach to SHG measurements was to compare the nonlinear signal intensity with an unpatterned patch, much larger than the laser radius of approximately 1 μm in diameter. To compare with the SHG counts from the individual nanoantenna, with size smaller than the laser spotsize, we normalized the effective power on the sample by normalizing the values of the SHG counts, obtaining the SHG ratio, defined as:

$$SHG_{\text{ratio}} = \frac{I_{\text{ref}}}{A_{\text{laser}}} \frac{A_{\text{r}}}{I_{\text{r}}} \tag{1}$$

where I_{ref} is the SHG intensity collected from the reference sample, A_{laser} is the laser area, I_{r} the SHG intensity collected from the nanoantennas with radius r, and A_{r} is the relative hexagonal area. In Table I are reported the calculated hexagonal cross section area, and the relative ratio with the laser spot size area of 1 micron in diameter used in the estimation of the SHG ratio in the main text.

**Hexagon Radius (nm)**	**Hexagon Area (nm^2)**	**Relative Ratio**
260	175,902	0.224
270	189,122	0.241
280	202,857	0.258
290	217,109	0.276
300	231,878	0.295
310	247,163	0.315
320	262,964	0.335
330	279,281	0.356
340	296,115	0.377
350	313,465	0.399
360	331,331	0.422

TABLE I. Area of an hexagon for different radial size, defined as the distance from the center to a vertex, and the ratio with the area of a circle of 1 μm in diameter.

SUPPLEMENTARY NOTE VI: EVALUATION OF THE SHG CONVERSION EFFICIENCY AND NONLINEAR COEFFICIENT

The SHG conversion efficiency parameter, η_{SH} (extracted at 1 GW cm^{-2} intensity of the fundamental wavelength excitation), and the nonlinear peak coefficient, β_{SH}, are defined as follows:

$$\eta_{\mathrm{SH}} = \frac{\langle P_{\mathrm{SH}} \rangle}{\langle P_{\mathrm{FF}} \rangle} \qquad \text{and} \qquad \beta_{\mathrm{SH}} = \frac{\hat{P}_{\mathrm{SH}}}{\left(\hat{P}_{\mathrm{FF}}\right)^2}, \tag{2}$$

where $\langle P_{\mathrm{X}} \rangle$ and $\hat{P}_{\mathrm{X}}$ (X=SH, FF) represent the average and peak value of the power of the second harmonic (SH) or fundamental (FF) radiation, respectively. The relevant experimental details for extraction of the conversion efficiency parameters are provided in Tables II-V for the case of a nanoantenna with the maximum β_{SH} value, extracted from the experimental results under excitation with fundamental wavelength of 1350 nm. Figure S7 shows the detailed values of β_{SH} extracted as a function of nanoantenna radius and excitation wavelength.

Parameter	Fund. Wavel. [nm]	Laser Rep. Rate [kHz]	Pulse duration [fs]	FF average power [μW]	spot size [μm]	FF peak power [W]
Symbol	λ_{FF}	RR	τ_{p}	$\langle P_{\mathrm{FF}} \rangle$	w_0	$\hat{P}_{\mathrm{FF}}$
Formula	-	-	-	-	-	$\frac{\langle P_{\mathrm{FF}} \rangle}{RR \cdot \tau_{\mathrm{p}}}$
Value	1350	500	250	0.908	0.68	7.26

TABLE II. Experimental input parameters for fundamental wavelength at 1350 nm.

Parameter	Nanoantenna size [nm]	SH emitted photons [cts/s]	SH photon energy [eV]	SHG average power [pW]	SHG peak power [μW]
Symbol	r	I_{SH}	$E_{\mathrm{ph,SH}}$	$\langle P_{\mathrm{SH}} \rangle$	$\hat{P}_{\mathrm{SH}}$
Formula	-	-	$\frac{1239.84}{(\lambda_{\mathrm{FF}}/2)}$	$I_{\mathrm{SH}} \cdot E_{\mathrm{ph,SH}}$	$\frac{\langle P_{\mathrm{SH}} \rangle}{RR \cdot \tau_{\mathrm{p}}}$
Value	290	$1.02 \cdot 10^6$	1.84	0.3	2.4

TABLE III. Experimental results for the 290-nm-radius nanoantenna excited by fundamental wavelength of 1350 nm.

Parameter	Conversion efficiency at 1 GW/cm^2	Nonlinear Peak Coefficient [W^{-1}]
Symbol	η_{SH}	β_{SH}
Formula	$\frac{\langle P_{\mathrm{SH}}\rangle}{\langle P_{\mathrm{FF}}\rangle}$	$\frac{\hat{P}_{\mathrm{SH}}}{\left(\hat{P}_{\mathrm{FF}}\right)^2}$
Value	$3.3 \cdot 10^{-7}$	$4.56 \cdot 10^{-8}$

TABLE IV. Nonlinear parameters for the 290 nm radius nanoantenna excited by fundamental wavelength of 1350 nm.

Radius [nm]	I_{SH} [cts/s]	η_{SH} [10^{-6}]	β_{SH} [10^{-7}]
260	173686	0,056	0,78
270	450296	0,15	2,0
280	680005	0,22	3,0
290	1021880	0,33	4,56
300	815562	0,26	3,6
310	525735	0,17	2,3
320	232750	0,075	1,04
330	11345,1	0,0037	0,051
340	148890	0,048	0,66
350	217078	0,070	0,97
360	121054	0,039	0,54
REF. PAD	73100,0	0,024	0,33

TABLE V. SHG efficiency parameters for $\lambda_{\mathrm{FF}} = 1350$ nm for the nanoantennas (rows corresponding to radius value from 260 to 360 nm) and for the reference MoS_2/WS_2 hetero-bilayer (last row labelled *REF. PAD*).

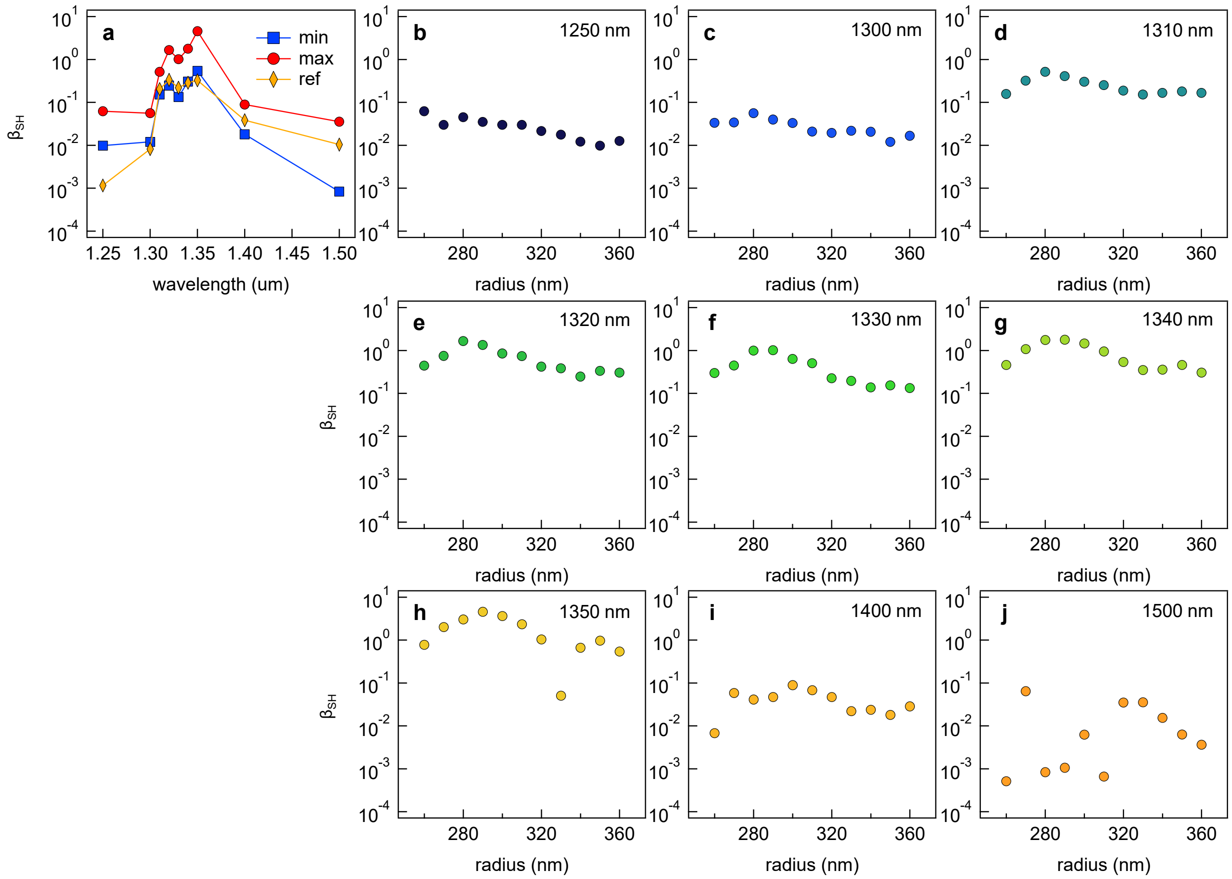

FIGURE S 7. Nonlinear peak coefficient β_{SH}. (a) Minimum and maximum value of the nonlinear peak coefficient β_{SH} (blu squares and red circles, respectively), calculated from the experimental results on the nanoantennas, as a function of the fundamental wavelength together with the β_{SH} coefficient calculated in the case of the reference MoS_2/WS_2 hetero-bilayer (yellow diamond-like markers). (b-l) Size-dependent coefficient β_{SH} for various fundamental wavelengths: (b) 1250 nm, (c) 1300 nm, (d) 1310 nm, (e) 1320 nm, (f) 1330 nm, (g) 1340 nm, (h) 1350 nm, (i) 1400 nm, and (j) 1500 nm.

SUPPLEMENTARY NOTE VII: LINEAR MICROSCOPY SETUP

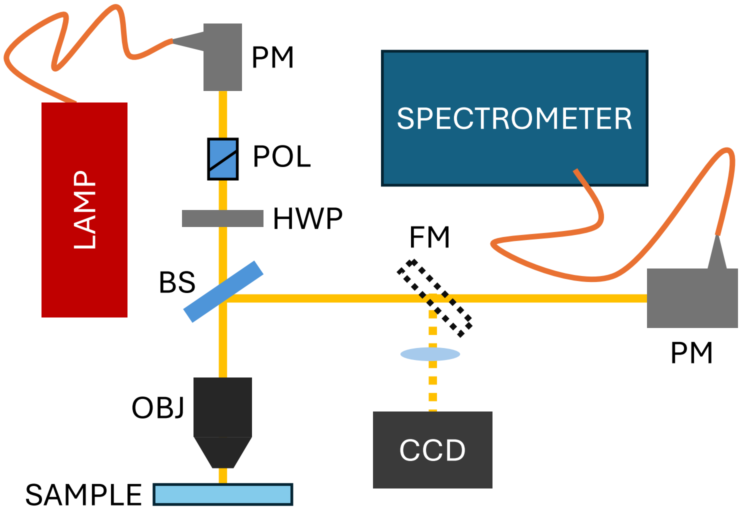

FIGURE S 8. Linear spectroscopy setup. PM: Parabolic mirror, POL: Polarizer, HWP: Half-wave plate, BS: Beam splitter, OBJ: objective, FM: Flip mirror.

SUPPLEMENTARY NOTE VIII: NONLINEAR SECOND HARMONIC GENERATION MICROSCOPY SETUP

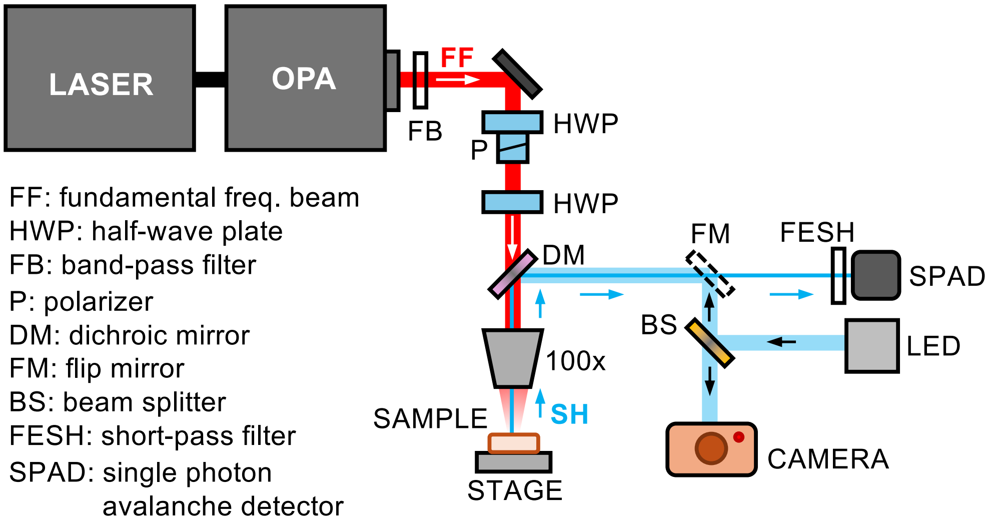

FIGURE S 9. Nonlinear spectroscopy setup.

* andrea.tognazzi@unipa.it; § These authors contributed equally to this work.
† paolo.franceschini@unibs.it; § These authors contributed equally to this work.
‡ luca.sortino@physik.uni-muenchen.de
